# Skills transfer from the DaVinci® system to the Hugo™ RAS system

**DOI:** 10.1007/s11255-023-03807-7

**Published:** 2023-09-29

**Authors:** Rikke Groth Olsen, Vladimir Karas, Flemming Bjerrum, Lars Konge, Hein Vincent Stroomberg, Julia Abildgaard Dagnæs-Hansen, Andreas Røder

**Affiliations:** 1 Copenhagen Prostate Cancer Center, Department of Urology, Copenhagen University Hospital - Rigshospitalet, Ole Malløes Vej 24, 2200 Copenhagen, Denmark; 2 Copenhagen Academy for Medical Education and Simulation (CAMES), Copenhagen, Denmark; 3 Faculty of Health and Medical Sciences, University of Copenhagen, Copenhagen, Denmark; 4 Department of Urology, Copenhagen University Hospital - Rigshospitalet, Copenhagen, Denmark; 5 Department of Gastrointestinal and Hepatic Diseases, Copenhagen University Hospital - Herlev and Gentofte, Herlev, Denmark; 6 Section of Biostatistics, Department of Public Health, University of Copenhagen, Copenhagen, Denmark

**Keywords:** Urology, Robot-assisted radical prostatectomies, Prostate cancer, Hugo™ RAS, IDEAL framework, Learning curve

## Abstract

**Purpose:**

Recently, the robotic surgical system, Hugo™ was approved for clinical use. The transfer of skills is important for understanding the implementation of surgical innovation. We explored the transfer of skills from the DaVinci® to the Hugo™ by studying the learning curve and short-term patient outcomes during radical prostatectomy (RARP).

**Methods:**

We examined the transfer of skills from one surgeon performing RARP from the first case with the Hugo™ system in April 2022. The surgeon had previously performed > 1000 RARPs using DaVinci®. Perioperative and clinical outcomes were collected for procedures on both Hugo™ and DaVinci®. Patient follow-up time was 3 months.

**Results:**

Nineteen Hugo™ cases and 11 DaVinci® cases were recorded. No clinically relevant difference in procedure time was found when transferring to Hugo™. Patients operated using Hugo™ had more contacts postoperatively compared to the DaVinci®, all Clavien–Dindo (CD) grade 1 (53% vs 18%). Three patients from the Hugo™ group were re-admitted within 30 days with catheter malfunction (CD grade 1), infection without a focus (CD grade 2), and ileus due to a hernia in the port hole (CD grade 3b). The 3-month follow-up showed similar results in prostate-specific antigen levels (PSA) and erectile dysfunction between the two robotic systems, but a higher incidence of incontinence was found for the Hugo™.

**Conclusion:**

We observed that the skills of an experienced robotic surgeon are transferable from DaVinci® to Hugo™ when performing RARP. No obvious benefits were found for using Hugo™ compared to DaVinci® for RARP although this needs confirmatory studies.

**Supplementary Information:**

The online version contains supplementary material available at 10.1007/s11255-023-03807-7.

## Introduction

Minimally invasive surgery for prostate cancer has evolved significantly in the twenty-first century with a transition from open surgery to the standard of care: robot-assisted surgery. The DaVinci® Surgical System has been the only system on the market since the introduction of robotic surgery but new robot-assisted systems are emerging. The Hugo™ RAS system was launched in late 2021. The safety and feasibility of the Hugo™ were documented in human cadaver studies and later in clinical studies in surgical urology [1–9]. Hugo™ differs from the DaVinci® system by having four individual arm carts, 3D glasses, and an open console [10].

Little is known about the transferability of skills between two robotic surgical systems. Transferability is important to understand as it can impact the resource needed for the hospital, the staff, and the surgeon in the implementation of new surgical robotic systems [11].

It is well known that there is a learning curve for surgeons adopting a new surgical modality, e.g., from open to robotic surgery, or learning a new surgical procedure, e.g., intraperitoneal to retroperitoneal nephrectomy [12, 13]. Learning curves describe the process of acquiring new skills with improvement in performance over time followed by a plateau where minimal, additional improvement is observed [14]. Previous research has shown that when surgeons transfer from open to robot-assisted radical prostatectomy there were higher incidences of incontinence and positive surgical margins in the early part of the learning curve [15, 16]. We wanted to examine the effects of the implementation of Hugo™ at our hospital to ensure optimal quality and care for the patients. Therefore, we explored the learning curve and transfer of skills by examining short-term patient outcomes during and after robot-assisted radical prostatectomies (RARP) for an experienced DaVinci® RARP surgeon switching to Hugo™.

## Materials and methods

In April 2022, the Department of Urology, Rigshospitalet, Copenhagen University Hospital, started using the Hugo™. The implementation was done using the IDEAL framework stage 2a recommendations for reporting surgical innovation [17]. The focus was patient safety and the transfer of skills of the surgeon. The most experienced surgeon in our department with > 1000 RARP was chosen for the implementation process.

The surgeon completed a training program for the Hugo™ including simulator training, and dry-run training at Orsi Academy in Gent, Belgium, and Copenhagen Academy for Medical Education and Simulation (CAMES), Rigshospitalet, Denmark.

From April 2022 to November 2022, we collected data on all RARPs performed on both the first Hugo™ RARP and the DaVinci RARPs performed in this period. One experienced RARP surgeon performed all surgeries. Due to technical issues and staff shortages, only a limited number of RARPs were performed on the Hugo™ in the period.

Data were registered with an on-site observer from the observation team. The team consisted of three different observers who all received the same training to register the on-site data. The procedure was registered from the time of the first incision (start time) until the time of skin closure (end time). Total time of port placement (from first incision until docking of the first arm), docking time (from port placement until the surgeon was at the console), console time, undocking, and skin closure were registered.

The console time was registered for the following part-procedures: lymph-node dissection (if performed), bladder-neck dissection (from dissection of the bladder until the beginning of the dissection of the seminal vesicles), removal of the prostate (from the start of dissection of seminal vesicles till the prostate was detached from the surrounding tissue), and the urethrovesical anastomosis (from the first till the last stitch).

After each procedure, the surgeon filled out three questionnaires about surgical performance and mental load. The Clinical record form of self-assessment of the individual RARP (CFR) assesses the surgeon’s satisfaction with their performance. It consists of 16 questions for both the difficulty of the cases and the performance satisfaction of each step (score 0–36, where a low score means few complications and high surgeon satisfaction) [18, 19]*.* The Surgery Task Load Index (SURG-TLX) analyses the mental load used to perform the surgery. It consists of six items on a continuous scale from 1 to 20 (total score range 6–120, where a low score means low mental load) [20]. The Technology Acceptance Model (TAM) assesses the acceptance of the new technology implemented in three domains: perceived usefulness, perceived ease of use, and change with a total of 16 questions (score 16–80, where a high score means high satisfaction with the equipment) [21].

Patient outcomes were collected from the electronic patient record and included peri- and postoperative data and surgical complications, according to Clavien–Dindo Classification (CD) [22], within the first 30 days (blood loss, length of hospital stay, re-admission, contact to the department) and at 3 months clinical follow-up (level of incontinence (continence is 0 pads/day), level of erectile dysfunction (patient-reported degree of erection (no-, partial, or total dysfunction), prostate-specific antigen (PSA)).

### Ethics

All patients signed an informed consent after receiving information about the study and the implementation process of the new surgical system. The study was reviewed and approved by The Danish Data Protection Agency (P-2022-341). No further approval was needed from the ethical committee as this was a qualitative assurance study.

### Statistical analysis

The total procedure time was adjusted for any technical malfunctions of the Hugo™ and the DaVinci® such as calibration problems, rebooting of the system, etc. The time spent on the technical malfunction was extracted from the affected part procedure. The total console time was adjusted for lymph-node dissection by extracting the time of lymph-node dissection from the total console time. This was done to standardize and compare the procedures. To explore the learning curve for the surgeon, we analyzed the data descriptively. The total times of the surgical part-procedures of Hugo™ and DaVinci® were compared by an independent sample t-test when normally distributed or otherwise by Wilcoxon signed-ranked test. Histograms were used to explore the normal distributions of the total times.

We used descriptive statistics to summarize the basic demographics and outcomes of the patients from the DaVinci® and the Hugo™ group. Categorical variables are described by frequencies and proportions while continuous variables are described by median and interquartile range.

SPSS (Version 28.0.; IBM SPSS Statistics for Windows, Armonk, NY) and R version 4.1.2 ((R Development Core Team, Vienna, Austria) running on RStudio version 2022.07.01 (© 2009–2022 by RStudio, Inc)) were used for the statistical analysis.

## Results

**Fig. 1:**
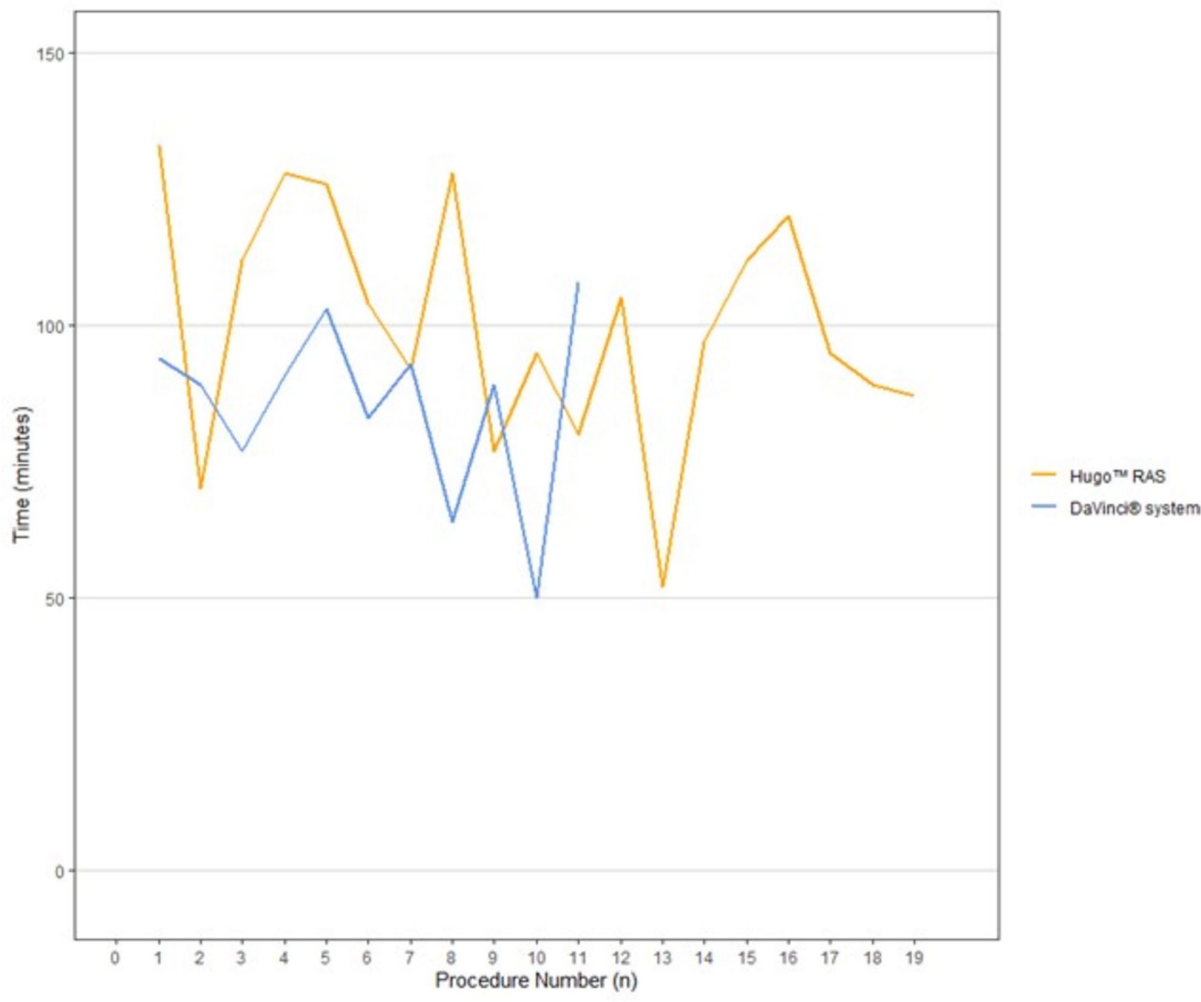
Correlation between procedure number and total console time for both the Hugo™ and the DaVinci® showing no clinically relevant improvement over time or difference between the systems

Compared to the DaVinci®, the median total console time adjusted for lymph-node dissection was a median 11 min longer for the Hugo™ (89 min vs 97 min, *p* = 0.06) (Fig. 1 and Table 1). The console time seems to decrease with a higher D’Amico cancer stage for both robotic systems (Supplementary Table 1). The bladder-neck dissection was 6 min shorter with Hugo™ compared with DaVinci (*p* = 0.03) (Table 1 and Supplementary Fig. 1).

**Table 1:** Intra-operative procedure times in minutes and post-surgery results with pathological outcome *p values < 0.05

	Hugo™	DaVinci®	*p* value
	Median [interquartile range]		
Console time^A^	97 [87–120]	89 [77–94]	0.06
Port placement	7 [6–8]	5 [5, 6]	0.39
Docking^A^	8 [6–11]	3 [2–5]	0.04*
Lymph-node dissection	65 [51–76]	67 [55–91]	0.61
Bladder-neck dissection	27 [19–35]	21 [18–25]	0.03*
Removal of the prostate	35 [30–51]	38 [25–44]	0.47
Urethrovesical anastomosis	33 [29–40]	29 [26–34]	0.11
Undocking^A^	3 [2, 3]	2 [1, 2]	0.04*
Skin closure^A^	10 [9–12]	8 [8–11]	0.19

	*n*	
Nerve-sparing		
No	8	1
Uni-lateral	5	6
Bilateral	6	4
Lymph-node dissection		
Yes	10	5
Metastasis?	5	2
No	9	6
Gleason score		
≤ 6	2	1
7	10	9
8–10	7	1
Positive surgical margins	7	3
Tumor stage		
pT2	11	9
pT3a	4	1
pT3b	4	1

^A^Tested by Wilcoxen signed-rank test due to non-normality, all others tested by independent *T* test

The time for docking of the robotic arms was 5 min faster for DaVinci® compared with Hugo™ (*p* = 0.04). Undocking time was 1 min faster for DaVinci® compared with Hugo™ (*p* = 0.04) (Table 1 and Supplementary Fig. 1).

Patients operated with the Hugo™ had a higher D’Amico cancer stage and a larger BMI range but were otherwise comparable to the patients operated on using the DaVinci® (Table 2).

**Table 2:** Pre-surgery demographics between the Hugo™ and the DaVinci®

	Hugo™	DaVinci®
Age, median in years [IQR]	66 [63–73]	59 [58–62]
BMI, median in kg/m^2^ [IQR]	25.5 [23.7–27.5]	26.1 [24.7–26.7]
ASA-score, median [IQR]	2 [2]	2 [1, 2]
Age-adjusted Charlson Comorbidity score, median [IQR]	2 [2, 3]	2 [1–3]
Former abdominal surgery, *n*		
Yes	6	1
No	13	10
Former TUR-P, *n*		
Yes	0	0
No	19	11
Prostate volume, median in ml [IQR]	47 [30–75]	43 [30–61]
PSA, *n*		
≤ 10	12	7
10–20	3	4
≥ 20	4	0
Gleason score, *n*		
≤ 6	4	4
7	9	6
8–10	6	1
Cancer stage, *n*		
≤ cT2a	10	8
cT2b	4	2
≥ cT2c	5	1
D’Amico cancer stage, n		
Low-risk	3	3
Intermediate-risk	7	7
High-risk	9	1

*IQR* interquartile range, *BMI* body mass index, *ASA-score* American Society of Anaesthesiologists score, *TUR-P* transurethral resection of the prostate, *PSA* prostate-specific antigen

All procedures were completed successfully with no perioperative complications (Table 3), and patients were discharged the day after surgery. Two patients from the Hugo™ group experienced minor postoperative complications during their hospital admission, both CD grade 1. The same were found for the DaVinci® group. Patients from the Hugo™ group had more contacts with the Department of Urology within the first 30 days than the DaVinci® group, all CD grade 1 (53% vs 18%). Three patients from the Hugo™ group were re-admitted to the Department of Urology within 30 days with catheter malfunction (CD grade 1), infection without a focus treated with IV antibiotics (CD grade 2), and ileus due to a hernia at the port hole treated with diagnostic laparoscopy and IV antibiotics (CD grade 3b). For the Hugo™ group, a higher incidence of complications was found in patients in the intermediate-risk group (*n* = 6) and high-risk group (*n* = 10) than in the low-risk group (*n* = 1) (Supplementary Table 1).

**Table 3:** 30-day- and 3-Months follow-up with surgical complications, incontinence rates, and erectile dysfunction

	Hugo™	DaVinci®
30-day follow-up		
Blood loss, median in ml [IQR]	300 [150–400]	200 [100–350]
Length of hospital stay, median in days [IQR]	1 [1]	1 [1, 2]
Conversion to open or laparoscopic, n	0	0
In-hospital complications, *n*		
CD Grade 1	2	2
CD Grade ≥ 2	0	0
Contact to the Department of Urology, *n*		
CD Grade 1	12	1
CD Grade ≥ 2	0	1
Re-admission, *n*		
CD Grade 1	1	0
CD Grade ≥ 2	2	0
3-months follow-up		
PSA, median in µg/L [IQR]	0 [0–0.45]	0 [0]
Incontinence, *n*		
Yes	7	1
No nerve-sparing performed	3	1
No	11	9
Unknown	1	1
Erectile dysfunction, *n*		
No	5	2
Partial	4	3
No nerve-sparing performed	1	0
Total	7	4
No nerve-sparing performed	4	0
Unknown	3	2

*IQR* interquartile range, *CD* Clavien–Dindo, *PSA* prostate-specific antigen

At 3-month follow-up, patients in the two groups had similar results regarding PSA levels and patient-reported erectile dysfunction (Table 3). A higher number of patients reporting incontinence was seen for the Hugo™ group (*n* = 7) compared to the DaVinci® group (*n* = 1).

**Fig. 2:**
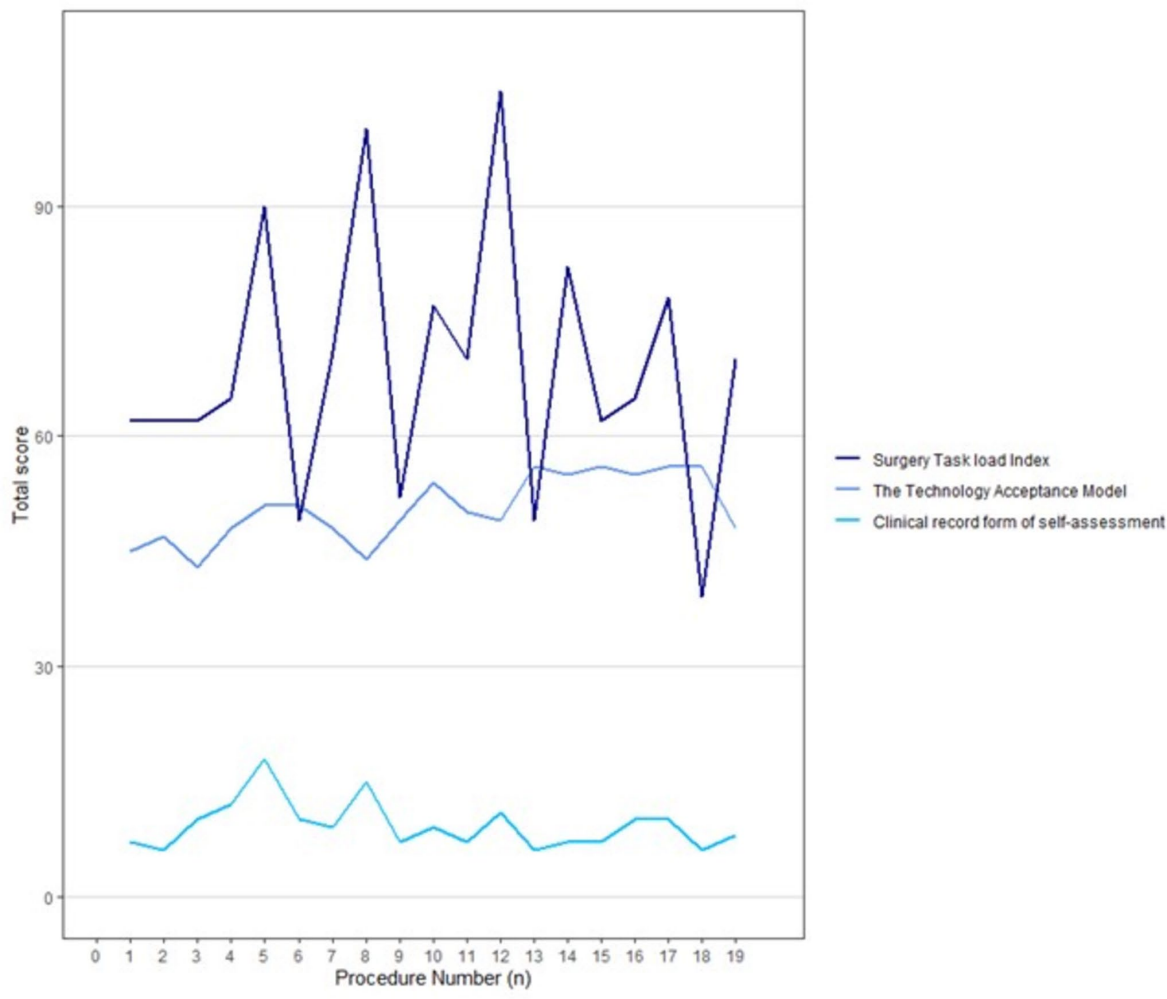
The progression of the total score of mental load and satisfaction of the surgeon over time for the Hugo™ system

There was little change over time in the mental load (SURG-TLX) and surgical satisfaction of the surgeon (CFR) during the experience with the Hugo™ (Fig. 2). A higher mental load was registered for the Hugo™ with 12.5 points (*p* = 0.07) higher than the DaVinci®. The acceptance of the robotic system (TAM) increased with the use of the Hugo™ from 43 to 56 points.

## Discussion

Since the first RARP in 2000, DaVinci® has transformed urology and today many consider DaVinci® procedures as the gold standard for several urological conditions [23]. After a longstanding monopoly for DaVinci®, new robotic systems are now put into clinical use. It is of particular interest if the technical innovation and development offer new improvements for the users and/or the patients. The arrival of the Hugo™ has been awaited with great expectation as it has a focus on wristed instruments, better ergonomics, and enhanced 3D imaging to allow the surgeon greater precision and control. However, with new equipment comes new learning curves and we aimed to explore this implementation process from the user’s experience.

Overall, we demonstrated that an experienced robotic surgeon could successfully switch from the DaVinci® to the Hugo™ in RARP surgery without a clinically relevant performance dip. We did not observe any improvements in surgical time or patient outcomes in this implementation phase. It seems that both console time and docking of the system are unfavorable for Hugo™ when starting to use the system even though the clinical implications seem small. This is similar to the results by Bravi et al. [1] and Ragavan et al. [8] who looked into the initial experience of using the Hugo™ for RARP.

Surprisingly for the 30-day follow-up, we observed more inquiries and re-admissions to the Department of Urology for patients who underwent surgery with the Hugo™ compared to the DaVinci®. One patient had a CD grade 3b complication due to the quality of the closure of the trocar access causing mechanical ileus. The complications observed in our study were not related to the robotic system itself and have previously been described with the DaVinci® [24]. Bravi et al. [1] showed similar complication reasons for re-admissions. We found patients undergoing surgery with Hugo™ had a higher D’Amico cancer stage and a larger BMI range. Whether this difference in complications is real or due to increased awareness from the patients and the department because of the implementation of the new Hugo™ can only be speculative. Our study is underpowered to answer that question and the finding may be random.

At 3-month follow-up, we found similar results for Hugo™ and DaVinci®. Postoperative PSA levels, positive surgical margins, and erectile dysfunction suggest there is a very short learning curve for the surgeon. We did, however, find a higher level of incontinence for the Hugo™ group. A bigger part of the patients operated on using the Hugo™ were high-risk cancer patients and, consequently, more patients in this group had either no nerve-sparring or uni-lateral nerve-sparring which could be an explanatory factor. As both incontinence and erectile dysfunction can improve for up to a year post-surgery [25], it is too early to conclude if there are differences.

For the surgeon, Hugo™ seemed to require a greater mental load throughout the surgical procedure even though the surgeon got more acquainted with the system over time. Our surgeon continued to perform surgery on the DaVinci® while transitioning to the Hugo™ and could compare the two systems head-to-head. The surgeon has used the DaVinci® for decades and has gotten used to a certain way of working. As a surgical community, we also need to address if surgeons should be switching between different robotic systems as this will demand the re-invention of the robotic educational programs to accommodate multi-platform training. The Hugo™ has the advantage of an open console, which allows for better supervision compared to the older DaVinci® X and S systems. The experienced surgeon has the same surgical view as the novice surgeon in the open console [26]. The experienced surgeon can easier guide the novice surgeon and it is easier for the surgeon to take over the console for demonstration purposes. Robotic systems with open consoles could, therefore, be a turning point in teaching robotic skills to novice surgeons and this could be the major contribution of Hugo™ to the new robotic technical era. We are still far from knowing the future benefits and strengths of each surgical robotic system but expectations are high with the launch of the new robotic systems. More comparative studies between robotic surgical systems, and randomized controlled trials are needed to explore the benefits and downsides of each new system. However, the ongoing development of new robotic systems may spark new technological possibilities.

The main limitation of our study is that it was an experienced single-surgeon study from one center. It is unknown how less experienced surgeons will perform. This should be investigated further. Furthermore, the small sample size limits the interpretation of patient outcomes in both the Hugo™ group and the DaVinci® group. We did not explore the impact of the implementation process on the hospital level or the overall economy which is of interest to the payer.

We observed that the skills of an experienced robotic surgeon are transferable from DaVinci® to Hugo™ when performing RARP. No obvious benefits were found for using Hugo™ compared to DaVinci® for RARP although this needs confirmatory studies.

### Supplementary Information

Below is the link to the electronic supplementary material.

Supplementary Figure 1 Overview of the time of different parts of the surgical procedures between the Hugo™ and the DaVinci® systems. A) Port placement, B) Docking, C) Bladder-neck dissection, D) Removal of the prostate, E) Urethrovesical anastomosis, F) Undocking, G) Skin closure (JPG 41 KB)

Supplementary Table 1: The total console time and total incidence of complications based on the D’Amico cancer stage and robotic platform (DOCX 17 KB)

## Data Availability

Data available on request from the authors and participant consent.

## Abbreviations

RARP: Robot-assisted radical prostatectomy
CFR: Clinical record form of self-assessment
SURG-TLX: Surgery Task Load Index
TAM: The Technology Acceptance Model
CD: Clavien–Dindo
