# Supplementary figures and images for: Skills transfer from the DaVinci® system to the Hugo™ RAS system
Source: Int Urol Nephrol. 2023 Sep 29;56(2):389–97. doi: 10.1007/s11255-023-03807-7 (PMC10808529; doi:10.1007/s11255-023-03807-7)

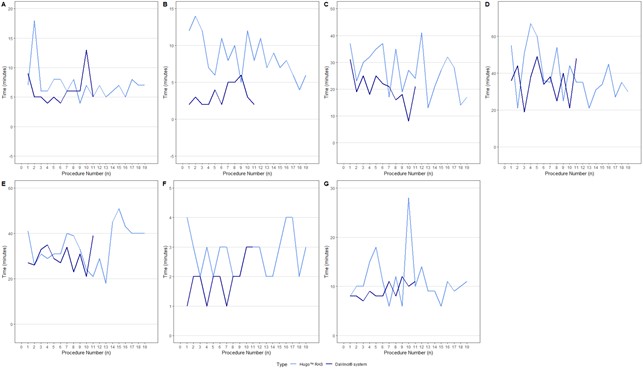

Supplement: Supplementary file 1 — Supplementary Figure 1 Overview of the time of different parts of the surgical procedures between the Hugo™ and the DaVinci® systems. A) Port placement, B) Docking, C) Bladder-neck dissection, D) Removal of the prostate, E) Urethrovesical anastomosis, F) Undocking, G) Skin closure (JPG 41 KB) [file 11255_2023_3807_MOESM1_ESM.jpg]
