# Supplementary material for: Skills transfer from the DaVinci® system to the Hugo™ RAS system
Source: Int Urol Nephrol. 2023 Sep 29;56(2):389–97. doi: 10.1007/s11255-023-03807-7 (PMC10808529; doi:10.1007/s11255-023-03807-7)
Supplement: Supplementary file 2 — Supplementary Table 1: The total console time and total incidence of complications based on the D’Amico cancer stage and robotic platform (DOCX 17 KB) [file 11255_2023_3807_MOESM2_ESM.docx]

**SUPPLEMENTARY TABLE 1**

|  | **D’Amico cancer stage** | **Console Time,** *median in minutes (range) or [IQR]* | **Total incidence  of complications,** *n* |
| --- | --- | --- | --- |
| **Hugo™** |  |  |  |
|  | *Low-risk (n=3)* | 120 (112-128)^ | 1 |
|  | *Intermediate-risk (n=7)* | 112 [95-128] | 6 |
|  | *High-risk ( n=1)* | 87 [74-96] | 10 |
| **DaVinci®** |  |  |  |
|  | *Low-risk (n=3)* | 91 (89-103)^ | 1 |
|  | *Intermediate-risk (n=7)* | 89 [77-94] | 2 |
|  | *High-risk (n=9)* | 50 | 1 |
|  |  |  |  |

^Range reported due to the low number in the group
